# Prediction Model for 30-day Outcomes Among Emergency Department Patients with Lower Gastrointestinal Bleeding

**DOI:** 10.5811/westjem.2020.1.45420

**Published:** 2020-02-24

**Authors:** Rosa Ramaekers, Jeffrey Perry, Cameron Leafloor, Venkatesh Thiruganasambandamoorthy

**Affiliations:** * Ottawa Hospital Research Institute, The Ottawa Hospital, Ottawa, Ontario; † University of Ottawa, School of Epidemiology, Epidemiology and Public Health, Ottawa, Ontario; ‡ University of Ottawa, Department of Emergency Medicine, Ottawa, Ontario

## Abstract

**Introduction:**

There are currently no robust tools available for risk stratification of emergency department (ED) patients with lower gastrointestinal bleed (LGIB). Our aim was to identify risk factors and develop a preliminary model to predict 30-day serious adverse events among ED LGIB patients.

**Methods:**

We conducted a health records review including adult ED patients with acute LGIB. We used a composite outcome of 30-day all-cause death, recurrent LGIB, need for intervention to control the bleeding, and severe adverse events resulting in intensive care unit admission. One researcher collected data for variables and a second researcher independently collected 10% of the variables for inter-observer reliability. We used backward multivariable logistic regression analysis and SELECTION=SCORE option to create a preliminary risk-stratification tool. We assessed the diagnostic accuracy of the final model.

**Results:**

Of 372 patients, 48 experienced an adverse outcome. We found that age ≥75 years, hemoglobin ≤100 g/L, international normalized ratio ≥2.0, ongoing bleed in the ED, and a medical history of colorectal polyps were statistically significant predictors in the multivariable regression analysis. The area under the curve (AUC) for the model was 0.83 (95% confidence interval, 0.77–0.89). We developed a scoring system based on the logistic regression model and found a sensitivity 0.96 (0.90–1.00) and specificity 0.53 (0.48–0.59) for a cut-off score of 1.

**Conclusion:**

This model showed good ability to differentiate patients with and without serious outcomes as evidenced by the high AUC and sensitivity. The results of this study could be used in the prospective derivation of a clinical decision tool.

## INTRODUCTION

Acute lower gastrointestinal bleeding (LGIB) is a common presentation to the emergency department (ED) and requires hospitalization for up to 87 per 100,000 adults per year.^1^–^3^ Mortality from LGIB can be up to 5%.^4^–^10^ Further, 20% will have recurrent bleeding while being hospitalized after a first LGIB episode,^7^,^8^,^11^,^12^ and as many as 24% of patients require an intervention to control the hemorrhage.^4^–^8^,^11^ Endoscopy plays a major role in the management of LGIB patients; however, there are limited resources for safe after-hours endoscopy in Canada.^13^ Risk-stratification of LGIB patients in the ED could help identify patients who need urgent intervention and those who could be safely managed as outpatients.

While several risk tools have been developed, most are not applicable to ED patients as they only include admitted patients and exclude patients who are discharged. Further, these studies have small patient cohorts, include a large number of predictor variables in their final model, and have a low diagnostic accuracy.^8^,^9^,^14^–^19^ Emergency physicians need a new decision tool that overcomes these limitations and will aid them in making evidence-based disposition decisions. The objective of this study was to identify risk factors for serious outcomes among ED patients with LGIB and to develop a preliminary model for a risk-stratification tool to predict 30-day adverse events.

## METHODS

### Study Design

This was a retrospective cohort study approved by the Ottawa Health Science Network Research Ethics Boards. The research ethics boards of the Queensway-Carleton Hospital approved the study protocol for health records review for patient follow-up and outcome ascertainment.

### Study Setting and Population

We conducted the study at two tertiary-care EDs of the Ottawa Hospital among adult patients who presented with acute LGIB between August 2013–June 2014. Clinically, an acute LGIB was defined as bright red blood per rectum in the prior two days, bright red blood on the glove after digital rectal examination, or a clear red bloody stool during the ED visit. We identified potential eligible patients using *International Classification of Disease*, 10^th^ Revision, codes related to LGIB. We excluded patients with the following characteristics: evidence of an upper gastrointestinal bleed without signs of a LGIB; patients who were already hospitalized; those designated palliative with less definitive interventions offered; LGIB secondary to a trauma; and patients who were not from the local area. We excluded multiple patient visits and only included the first visit for LGIB-related symptoms to the ED.

### Study Protocol

One investigator collected variables and outcomes using a standardized case record form. A second investigator collected data for 10% of a random selection of the total patient cohort. We calculated a k-value for inter-observer reliability.

### Outcomes

The primary outcome was a 30-day composite outcome of all-cause mortality; significant rebleeding; an intervention to manage the bleeding; and intensive care unit (ICU) admission. Patients could experience multiple outcomes. We defined recurrent bleeding as a significant rebleeding (drop in hemoglobin, requiring blood transfusion or readmission) after 24 hours of clinical stability that was objectively identified on physical examination or endoscopy after index visit disposition. Need for intervention was defined as receiving any of blood transfusion, undergoing endoscopic or surgical intervention, or having angiographic embolization to control the bleeding after the index visit.

**Population Health Research Capsule**

What do we already know about this issue?

There are no robust risk tools to predict 30-day adverse outcomes for emergency department (ED) patients with lower gastrointestinal bleed (LGIB).

What was the research question?

We sought to identify risk factors and develop a preliminary model to predict 30-day adverse outcomes in ED LGIB patients.

What was the major finding of the study?

We developed a preliminary model with five predictors that identifies ED LGIB patients at low-risk for 30-day adverse outcomes.

How does this improve population health?

Risk-stratification of ED LGIB patients could reduce the burden on the healthcare system and the associated healthcare costs.

### Data Analysis

We calculated a sample size of 376 patients, based on a sensitivity of 95% and a bound on error of estimation between 4%–5%.^20^ In order to include predictors in our preliminary model, we excluded variables with >25% of data missing, a cell count of ≤5, or a p-value >0.20 in the univariate analysis. We dichotomized the remainder of the variables based on clinical relevance and statistical significance. We then proceeded with multiple imputation with 10 datasets to account for missing data for variables with <25% missing data. We tested for collinearity and removed variables based on clinical and statistical significance.

We performed logistic regression to identify risk factors and derive a preliminary model. We used a stepwise backward selection method with a p-value of <0.01 and the SELECTION=SCORE function in SAS software 9.4 (SAS Institute Inc., Cary, NC, USA). This method produces a model with a specific number of variables, which is useful when the number of outcomes is limited. We used Rubin’s rules to combine the estimates of the datasets from the multiple imputation database.^21^

We developed a scoring system based on the point estimates of the odds ratios for the variables in the logistic regression analysis. Variables with a point estimate between 0.5 and 1.49 were assigned one point in the scoring system, and variables with a point estimate between 1.5 and 2.49 were assigned two points. We then calculated sensitivity, specificity, positive predictive value (PPV), negative predictive value (NPV), positive likelihood ratio (LR+), negative likelihood ratio (LR−) and area under the curve (AUC) with 95% confidence intervals (CI) per cut-off score.

## RESULTS

We identified 766 potential eligible patients and included 372. See the Figure for a flow diagram. Outcomes were as follows: 61 patients (16.4%) suffered a serious outcome within 30 days; 13 outcomes occurred in the ED (2.5%), while 48 (12.9%) happened after ED disposition. Four patients died, 12 experienced a recurrent bleeding, and 47 interventions were performed to stop the bleeding.

Despite that the INR was only measured in 200 patients (53.8%), we reasoned that it was a clinically relevant variable and decided to use multiple imputations to deal with the missing data. We calculated the mean international normalized ratio (INR) for patients who were taking anticoagulants or diagnosed with liver cirrhosis (mean INR >2.0) and imputed this mean for patients with a missing INR who were on anticoagulants or had liver cirrhosis. We did the same for patients who were not on anticoagulants and did not have a liver disease (mean INR <2.0).

Using the SELECTION=SCORE option in SAS on the 10 datasets from the multiple imputation database, we found that the following five predictors were statistically significant in all 10 datasets: age ≥75 years; INR ≥2.0; hemoglobin ≤100 grams per liter (g/L); clear red bloody stool in the ED; and past medical history of colorectal polyps. These results were similar using the backward selection method.

We then assigned points to the variables based on the point estimates of the odds ratio derived from the logistic regression analysis (Appendix 1). Patients with a hemoglobin ≤100 g/L or INR ≥2.0 were assigned two points for each positive variable. Patients ≥75 years, who had a clear red bloody stool in the ED, or a past medical history of colorectal polyps were assigned one point for each positive variable. This added up to a maximum of seven points. When using a cut-off score of one, the sensitivity was 0.96 (95% CI, 0.90–1.00) and specificity was 0.53 (95% CI, 0.48–0.59). The sensitivity decreased and specificity increased with a higher cut-off score. The AUC was 0.83 (95% CI, 0.77–0.89) (Appendix 2). See the table for the classification performance of all possible cut-off scores.

## DISCUSSION

We found five predictors that could aid in risk-stratification of ED LGIB patients: age ≥75 years; INR ≥2.0; hemoglobin ≤100 g/L; clear red bloody stool in the ED; and a past medical history of colorectal polyps. This model had a very good AUC (0.83, 95% CI, 0.77–0.89). The sensitivity and negative predictive value for a cut-off score of one are high, while the negative likelihood ratio is close to zero. Clinically, this means that patients with no risk factors are at very low risk to experience an outcome (Appendix 3). Therefore, this score could aid emergency physicians in identifying low-risk patients who can be managed in an outpatient setting, which could reduce the burden and associated costs on the healthcare system.

This score is an objective addition to clinical gestalt, as clinical gestalt is often influenced by patient-specific and physician-specific factors. As with all clinical decision rules, clinicians will use their judgment as to whether extenuating circumstances place a given patient at particularly high risk for a poor outcome despite the score placing them at low risk, or vice versa. Our priority was to develop a score that would identify low-risk patients, as we thought this would be most valuable to emergency physicians. However, we do acknowledge that ideally a risk score should identify both low-risk and high-risk patients, and all diagnostic accuracies should be high.

## LIMITATIONS

The data were retrospectively collected, which is not optimal for establishing a risk prediction model. However, most of the risk factors identified are reliably recorded, clearly understood clinical variables, which minimizes this limitation. We dealt with missing data by excluding variables with >25% missing data, and by using multiple imputations for variables with <25% missing data. INR had >25% missing data; however, we did not exclude it because it was thought to be a clinically relevant variable. We think INR was missed in so many patients as it is not always initially drawn and sometimes has to be added on to the blood work later. A future derivation study should be prospective with robust data collection to reduce the proportion of patients with missing data.

Another limitation is that we only had 48 adverse outcomes, which limits the number of predictors we could include in the final model. This is acceptable as it is a preliminary model, but a future study should include more patients and more adverse outcomes. Further, we used a composite outcome of death, recurrent bleeding, need for intervention, and ICU admission. This was based on previous GI bleed risk-stratification studies and allowed us to identify all high-risk and low-risk patients. We did not compare our data to clinical gestalt as our data were retrospectively collected. Future prospectively collected studies should compare the use of the risk score to clinical gestalt.

## CONCLUSION

In this retrospective cohort study, we identified five predictors that could aid in the risk-stratification of ED LGIB patients: age ≥75 years, INR ≥2.0; hemoglobin ≤100 g/L; clear red bloody stool in the ED; and a past medical history of colorectal polyps. Patients with high-risk criteria should be considered for timely management. Future, multicenter, prospective studies should be done to confirm our results, to externally validate the score, and to study the implementation of the score in clinical practice.

## Supplementary Information







## Figures and Tables

**Figure:**
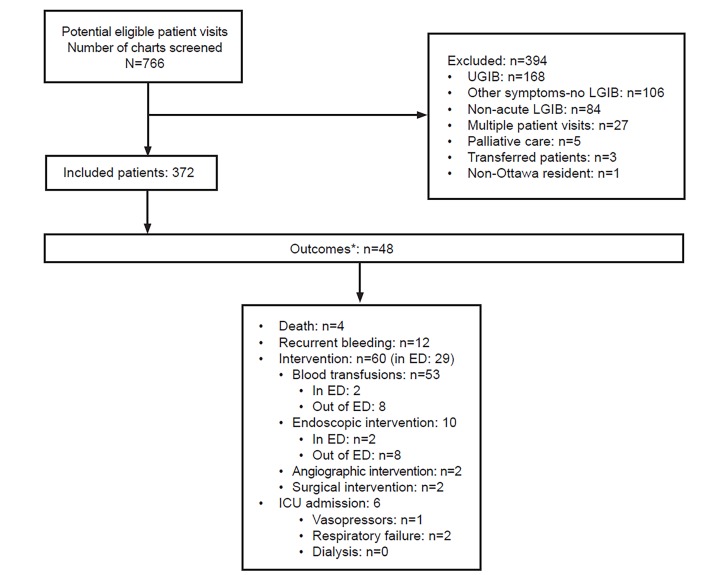
Flow diagram of patient selection. *Outcomes after ED disposition. Patients can experience multiple outcomes. *UGIB*, upper gastrointestinal bleeding; *LGIB*, lower gastrointestinal bleeding; *ED*, emergency department; *ICU*, intensive care unit.

**Table:** Diagnostic accuracies of the prediction model per cut-off score.

Cut-off scores*	Sensitivity (95% CI)	Specificity (95% CI)	PPV (95% CI)	NPV (95% CI)	Positive LR (95% CI)	Negative LR (95% CI)
1	0.96 (0.90–1.00)	0.53 (0.48–0.59)	0.23 (0.17–0.29)	0.99 (0.97–1.00)	2.06 (1.80–2.34)	0.08 (0.02–0.30)
2	0.69 (0.55–0.82)	0.76 (0.70–0.81)	0.34 (0.25–0.44)	0.93 (0.89–0.97)	2.84 (2.11–3.82)	0.41 (0.26–0.64)
3	0.49 (0.35–0.63)	0.91 (0.86–0.94)	0.49 (0.35–0.63)	0.91 (0.86–0.94)	4.93 (3.06–7.97)	0.57 (0.43–0.75)
4	0.33 (0.20–0.47)	0.98 (0.97–1.00)	0.73 (0.54–0.91)	0.91 (0.88–0.94)	18.00 (7.41–44.00)	0.68 (0.56–0.83)
5	0.11 (0.00–0.23)	0.99 (0.98–1.00)	0.60 (0.17–1.00)	0.93 (0.89–0.95)	16.00 (2.80–92.00)	0.90 (0.78–1.02)
6	0.03 (0.00–0.06)	1.00 (1.00–1.00)	1.00 (1.00–1.00)	0.87 (0.84–0.91)	Inf 0.82–482.00	0.98 (0.92–1.02)
7†						

* The diagnostic accuracies are reported for the value of the cut-off score or higher

† 0 patients had a score of 7

*CI*, confidence interval; *PPV*, positive predictive value; *NPV*, negative predictive value; *LR*, likelihood ratio.
