# Supplementary material for: Prediction Model for 30-day Outcomes Among Emergency Department Patients with Lower Gastrointestinal Bleeding
Source: West J Emerg Med. 2020 Feb 24;21(2):343–7. doi: 10.5811/westjem.2020.1.45420 (PMC7081856; doi:10.5811/westjem.2020.1.45420)
Supplement: Supplementary file 1 [file wjem-21-343-s001.docx]

**Appendix 1.** Five variables included in the final model with the point estimates and the amount of points each variable is assigned to in the final model.

| Variables | Point estimate | Points in model |
| --- | --- | --- |
| Hemoglobin ≤100 g/L | 2.061 | 2 |
| INR ≥2.0 | 1.530 | 2 |
| Age ≥75 years | 0.995 | 1 |
| Bloody stool in the ED | 1.237 | 1 |
| History of colorectal polyps | 1.321 | 1 |
