# Supplementary figures and images for: Prediction Model for 30-day Outcomes Among Emergency Department Patients with Lower Gastrointestinal Bleeding
Source: West J Emerg Med. 2020 Feb 24;21(2):343–7. doi: 10.5811/westjem.2020.1.45420 (PMC7081856; doi:10.5811/westjem.2020.1.45420)

**Appendix 2.** ROC curve for final model.


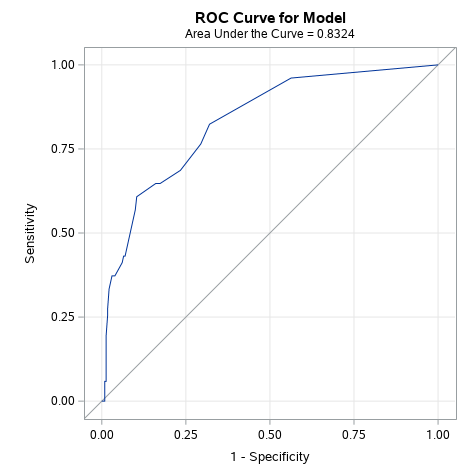

Supplement: Supplementary file 2 [file wjem-21-343-s002.docx]

**Appendix 3.** Clinical use of the risk score.


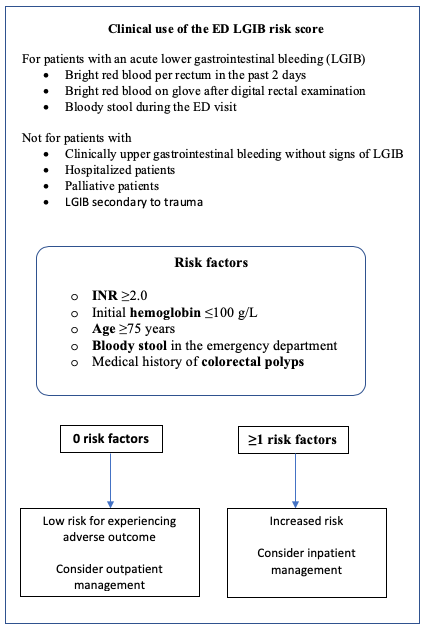

Supplement: Supplementary file 3 [file wjem-21-343-s003.docx]
